# Factors That Influence Patients’ Decisions About Repetitive Transcranial Magnetic Stimulation as a Treatment Option for Treatment-Resistant Depression: Protocol for a Prospective Mixed Methods Cohort Study

**DOI:** 10.2196/82491

**Published:** 2026-03-06

**Authors:** Alexandra Godinho, Sanvitti Dalmia, Steven Selchen, Daniel Tziatis, Pete Wegier

**Affiliations:** 1 Research Institute, Humber River Health, 1235 Wilson Avenue, North York, ON, M3M 0B2, Canada, 1 416-242-1000 ext 21203; 2 Department of Family and Community Medicine, University of Toronto, Toronto, ON, Canada; 3 Institute of Health Policy, Management and Evaluation, University of Toronto, Toronto, ON, Canada

**Keywords:** treatment-resistant depression, repetitive transcranial magnetic stimulation, mental health decision-making, shared decision-making, card sorting, mixed methods research

## Abstract

**Background:**

Treatment-resistant depression (TRD), affecting approximately 20% to 30% of individuals with major depressive disorder, is associated with significant disability, reduced quality of life, and an increased risk of hospitalization and suicide. Repetitive transcranial magnetic stimulation (rTMS), a noninvasive neuromodulation therapy, has demonstrated strong efficacy for TRD but is typically limited to research contexts or private clinics. Existing research on patient perspectives on rTMS is limited and largely retrospective, focusing on individuals who have already undergone treatment. As a result, little is known about the factors that influence patients’ decisions to accept or decline rTMS at the time of referral, particularly within real-world clinical settings.

**Objective:**

This study aims to address the gap in the literature by prospectively examining decision-making processes surrounding rTMS in a community hospital outpatient clinic.

**Methods:**

This prospective mixed methods cohort study will recruit 30 adults with TRD referred to a public rTMS clinic. Participants will be stratified based on their decision to opt in or out of treatment. Data collection will include hybrid card sorting interviews, self-report questionnaires (assessing depression, well-being, cognitive flexibility, decisional conflict, and health literacy), and medical chart reviews. Each participant will complete a baseline and 6-month follow-up interview and survey. Qualitative data will be analyzed using constant comparative analysis, informed by bounded rationality and prospect theory. Quantitative data will be analyzed using bivariate statistics and hierarchical cluster analysis to identify patterns in decision-making factors.

**Results:**

This study is being funded by a charitable donation from Jack and Pat Kay to Humber River Health, which is also supporting the establishment of the rTMS clinic, committed to in April 2024. Recruitment commenced in December 2025 and is expected to conclude in December 2026; no participants have been enrolled as of February 2026.

**Conclusions:**

To the best of our knowledge, this is the first study to prospectively examine decision-making regarding rTMS in a real-world, publicly funded clinic including both individuals who initiate and those who decline treatment. The findings may inform the development of patient educational and engagement materials and highlight gaps in patient-physician communication during the rTMS decision-making process.

## Introduction

Treatment-resistant depression (TRD) is a significant public health challenge, affecting approximately 20% to 30% of individuals with major depressive disorder (MDD) worldwide [1]. In Canada, more than 20% of primary care patients with MDD meet the minimum criteria for TRD, defined as the failure to respond to at least 2 adequate trials of antidepressant medication [2]. Furthermore, up to 55% of individuals with MDD may not achieve remission and could be diagnosed with TRD during their lifetime [3]. Individuals with TRD experience disproportionately high levels of disability, reduced quality of life, and an increased risk of hospitalization and suicide [4,5]. As a result, patients with TRD visit outpatient clinics 58% more frequently and have 3 times more psychiatric consultations compared with individuals with non-TRD MDD, placing a greater demand on health care resources [6]. These challenges underscore the need for improved access to more effective treatment strategies beyond traditional approaches such as medication switching or augmentation with psychotherapy [3]. One promising alternative is repetitive transcranial magnetic stimulation (rTMS), a noninvasive neuromodulatory intervention that has demonstrated efficacy in individuals with TRD [7,8].

Approved by multiple health care regulatory bodies such as the US Food and Drug Administration, European Medicines Agency, and Health Canada, rTMS uses focused magnetic field pulses to induce lasting changes in neural activity in brain regions associated with the regulation of thoughts, emotions, and behavior [9]. While conventional rTMS typically involves delivering high-frequency stimulation in 40-minute sessions over 4 to 6 weeks, a modified form known as intermittent theta-burst stimulation can be delivered in 3 to 8 minutes with comparable efficacy [10]. Despite these advancements, which improve accessibility through shorter treatment durations and increased capacity and cost-effectiveness, rTMS remains largely inaccessible to most individuals with TRD [11]. In Canada, rTMS is predominately offered through private clinics and research studies; however, the last 5 years have seen a growing number of public clinics offering rTMS through philanthropic donations.

To improve access to rTMS and ensure that new clinics understand the needs of patients, it is first crucial to understand patients’ perceptions, willingness, and likelihood of engaging with this treatment. The decision of individuals with TRD to pursue rTMS may be influenced by multiple factors, including knowledge of rTMS, family involvement, perceived efficacy, financial considerations, and trust in health care providers. A recent systematic review identified 4 studies examining psychiatric patient perceptions of noninvasive brain stimulation [12]. Overall, the review concluded that patients generally viewed rTMS positively, considering it to be as safe as antidepressant medication, recommending it to others, and reporting low levels of treatment-related fear. However, these studies offer limited insight into the decision-making process for rTMS. First, all 4 studies focused on patients after treatment, offering perspectives on the treatment process (eg, outcomes, side effects, and lasting changes) rather than perceptions of rTMS at the time of referral [13-16]. Second, only patients who had elected to receive rTMS were surveyed, leaving gaps in understanding the beliefs and/or barriers of those who declined treatment. Third, it is unclear whether participants in these studies were presented with a choice to pursue rTMS or whether it was the prescribed course of treatment for an acute inpatient group. Finally, all the studies relied solely on survey methods, which restrict a deeper exploration of the personal factors that influence patients’ decisions to accept or refuse rTMS treatment.

In the absence of studies directly examining treatment decision-making for rTMS, research on patient choices for receiving electroconvulsive therapy (ECT) offers a valuable comparative framework. This is particularly relevant given the limited public awareness and understanding of rTMS [17], with one study revealing that many individuals mistakenly associate rTMS with procedures and side effects more characteristic of ECT [17]. Among the few studies that have been conducted on patients receiving ECT, several key factors have been identified as influencing decisions, including trust in health care providers, the severity of depression, prior experience with and knowledge of ECT, feelings of helplessness, family support, and perceived stigma [18-21]. While similar factors may shape the decision-making process for individuals referred for rTMS, no studies to date have specifically investigated this.

This study aims to prospectively examine the factors influencing individual decision-making on opting in or out of rTMS for TRD in a community hospital clinic. Using a mixed methods approach (ie, card sorting interviews, surveys, and chart reviews), we will explore patients’ decision-making process at the time of referral and identify the key factors that influence their decisions. Additionally, we will examine how perceptions of the decision-making process evolve over time (ie, 6 months) and investigate individual-level characteristics such as demographic and clinical characteristics, cognitive flexibility, and general decision-making ability that may be associated with treatment decisions. The following research questions will be examined:

1. What factors (eg, trust, personal attitudes, practical barriers and accessibility, knowledge, past experiences, family and social relationships, culture, and religion) influence rTMS decision-making in patients diagnosed with TRD?
2. Do patients’ perceptions of key factors influencing rTMS decision-making change 6 months after their initial decision?
3. How do individual differences in cognitive flexibility, past depression treatment use, decisional conflict, and health literacy impact the decision to opt in or out of rTMS treatment?
4. How do patients perceive their role in the rTMS referral process, and what are their preferences regarding involvement and shared decision-making?

As this study is exploratory in nature, no a priori hypotheses have been made.

## Methods

### Participants

Outpatients at the Humber River Health (HRH) mental health and addictions clinic who have been referred for rTMS consultation by their treating psychiatrist will be invited to take part in a study examining factors influencing rTMS decision-making, including patient perceptions of involvement and shared decision-making. Eligibility criteria include being aged ≥18 years, having a diagnosis of TRD, receiving an rTMS referral from their treating psychiatrist, and being English speaking. Potential participants with contraindications for rTMS (eg, history of epilepsy or other seizure disorder, ferrous metallic implants in the skull, history of drug abuse within the previous 3 to 6 months, any unstable medical condition, or pregnancy or attempting to become pregnant) or cognitive impairments that prevent them from providing informed consent for treatment will be excluded. Exclusion criteria will be determined by the treating psychiatrist based on clinical judgment. It should be noted that the eligibility criteria for this study are the same as the eligibility criteria to receive rTMS treatment at HRH, with the exception of being English speaking, which will be determined by research staff upon initial contact.

### Study Design and Rationale

This project will be a prospective longitudinal cohort study using a concurrent mixed methods design to investigate key factors influencing rTMS decision-making in patients diagnosed with TRD. It will incorporate hybrid card sorting qualitative interviews, self-report surveys using validated scales, and medical chart reviews with a 6-month follow-up. Hybrid card sorting qualitative interviews were chosen as they are an interactive research methodology that encourages structured and accessible engagement with researchers [22]. The main analysis will include a quantitative component where cards will be grouped into clusters based on measurable attributes (eg, pile sorting and card rankings) and a qualitative constant comparative analysis (CCA) to explore the meaning that participants attach to each card when choosing whether to engage in rTMS. This dual approach will strengthen the depth and validity of the findings. Additionally, triangulating medical chart reviews will provide clinical context, offering further insights into how medical histories and treatment influence decision-making and vice versa. Finally, integrating quantitative survey data into the qualitative findings will allow for greater replicability, generalizability, and clinical utility by examining individual differences (eg, demographics and cognitive measures) that influence rTMS decision-making. This comprehensive approach will provide a more nuanced understanding of factors guiding patient decision-making for unfamiliar treatments such as rTMS.

### Recruitment and Study Procedures

Patients with TRD attending HRH’s outpatient mental health and addictions clinic may be referred to rTMS by their treating psychiatrist. rTMS will be available to clinically eligible patients free of cost as a result of a charitable donation to establish the new clinic; participants who do not enroll in the study will not be required to pay for treatment. The rTMS referral process will involve three steps: (1) discussing rTMS as an option with their psychiatrist, (2) receiving a formal referral to the HRH rTMS clinic, and (3) attending an initial consultation at the rTMS clinic. Patients can opt out during steps 1 and 3 by declining rTMS or by not attending or canceling their initial consultation. Opting in will be defined as attending the initial consultation and scheduling their first rTMS session. A total of 30 participants will be recruited: 15 who opt in and 15 who opt out of rTMS.

Treating psychiatrists who identify patients as appropriate for and recommend rTMS (ie, informal discussion or prescription referral) will ask patients for consent to be contacted by the research institute regarding a study on treatment decision-making, regardless of their rTMS choice. This information will be documented on a file that is shared between clinicians and research staff on HRH’s institutional OneDrive (Microsoft Corp). This Microsoft Excel file will contain patient names and telephone numbers and will be destroyed upon recruitment. Research staff will contact consenting patients via telephone and invite them to participate in a study examining factors influencing rTMS decisions. For individuals who agree to an initial consultation, informed consent and study activities will be conducted subsequent to this appointment; as individuals may decline rTMS during the first consultation, this will ensure that an equal number of participants are recruited for the opt-in and opt-out groups.

Potential participants, regardless of rTMS decision, will receive an initial call from research staff to discuss the study and provide an opportunity for questions. Patients who would like more information can be sent an electronic copy of the consent form for further information via email. Informed consent will be scheduled to be obtained in person using teach-back strategies to ensure that potential participants make an informed choice to take part and understand that research activities are not part of the care they may receive while attending the clinic. Potential participants will be provided with up to 1 hour to discuss the contents of the consent form, and this discussion will be scheduled at the preference of the participant (ie, before or subsequent to a clinical appointment or on a separate occasion). Participants may decline to take part at any stage of the study and request to have their data withdrawn; however, only participants who consent to all study activities will be enrolled. In addition to obtaining informed consent to participate in the study, the consent process will include (1) optional consent to be contacted for future studies and (2) the option to be informed of study results at the close of the study. For clarity, consent to be contacted for future study interest will be collected separately. The results of the study will be distributed via email along with an optional survey on Microsoft Forms. This survey will consist of 4 questions designed to assess the accessibility, usefulness, and overall design of the summary. The questions will be derived from a modified version of the Information Assessment Method, which was originally developed to evaluate online health information for parents [23]. The collected feedback will be instrumental in refining and improving future participant-facing research materials, enhancing the overall communication strategy of our research efforts.

**Figure 1.**
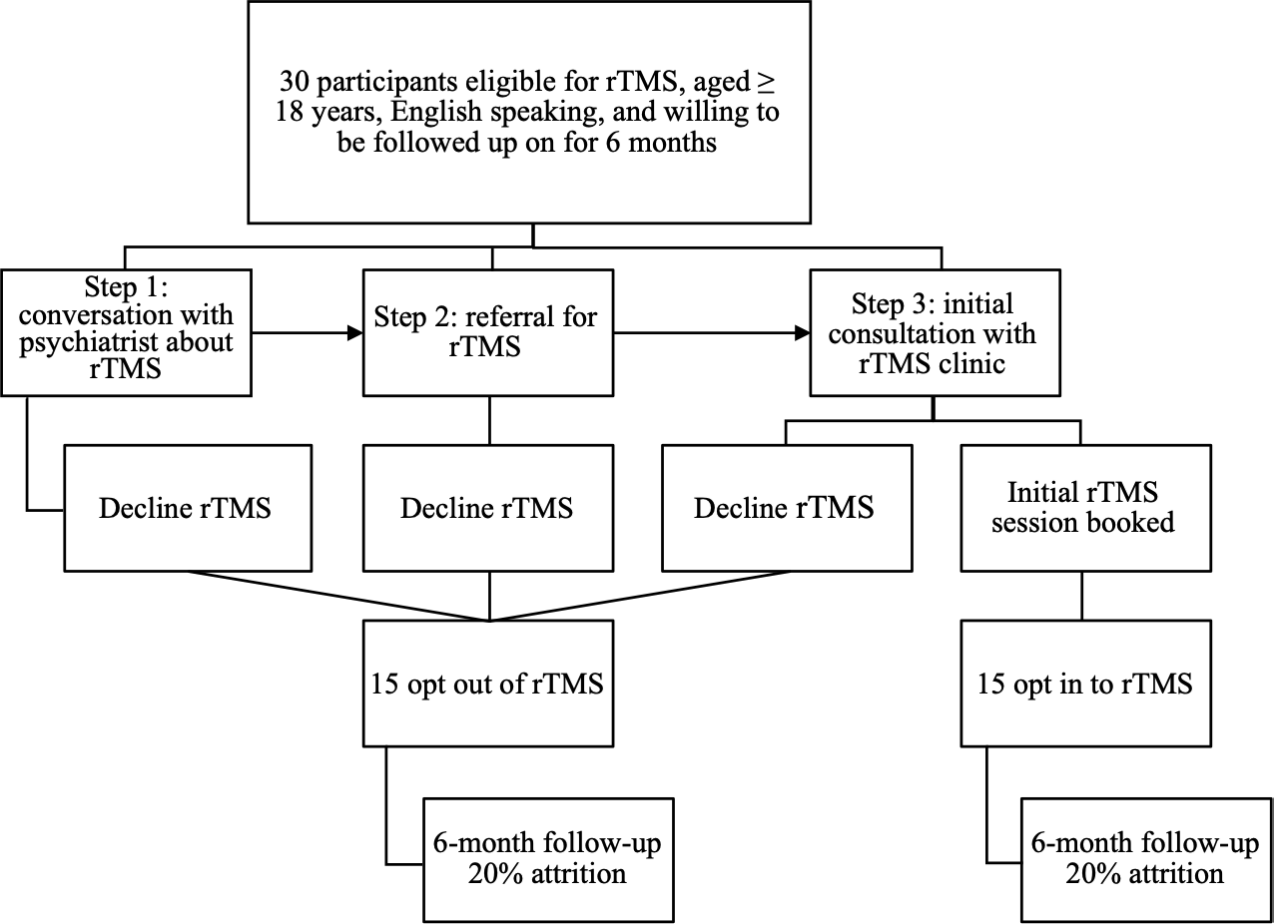
Overview of the proposed cohort study. rTMS: repetitive transcranial magnetic stimulation.

Participants who provide consent will be asked to complete a 1-hour in-person baseline card sorting interview and 30-minute survey. Six months after baseline, participants will be asked to schedule an additional in-person appointment to complete another 1-hour follow-up card sorting interview and 30-minute survey. Baseline and follow-up study activities can be completed over 1 or 2 appointments to accommodate participant schedules and minimize burden. Figure 1 provides the CONSORT (Consolidated Standards of Reporting Trials) diagram of the study.

### Ethical Considerations

This study was approved by the institutional research ethics board of our team’s hospital, HRH (25-0003), and all participants will provide informed written consent before being enrolled in the study. Participation in this survey is voluntary, and responses will not be linked to the participant study data or any other identifying information. All participants who complete the baseline card sorting interview and survey will be compensated with a CAD $35 (US $25.57) Walmart gift card, and those who complete the 6-month follow-up card sorting interview and survey will be compensated with an additional CAD $45 (US $32.88) Walmart gift card for a potential total of CAD $80 (US $58.46) for completing all study activities. Although the time commitment for each visit is the same, the increased honorarium at follow-up is intended to promote retention and reduce attrition, consistent with common practice in longitudinal research [24,25], and compensation amounts are in accordance with published guidelines for fair compensation of research participants (ie, CAD $25 [US $18.27] per hour) [26].

### Measures and Qualitative Methods

#### Hybrid Card Sorting Within Qualitative Interviews

A closed card sorting methodology incorporating “think-aloud” techniques [22,27,28] will be used to explore the factors influencing patient decision-making regarding rTMS treatment. The goal of the card sorting task will be to systematically identify and prioritize criteria that patients weigh when deciding to opt in or out of rTMS. A standardized deck of 24 cards has been developed with key factors relevant to decision-making for unfamiliar depression treatments such as rTMS. The content of the cards has been informed by an ongoing scoping review on individual-level factors that influence decision-making for depression treatment [29], including factors found relevant to ECT treatment choices [18-21], and by consultations with clinical staff of the HRH mental health and addictions clinic.

**Figure 2.**
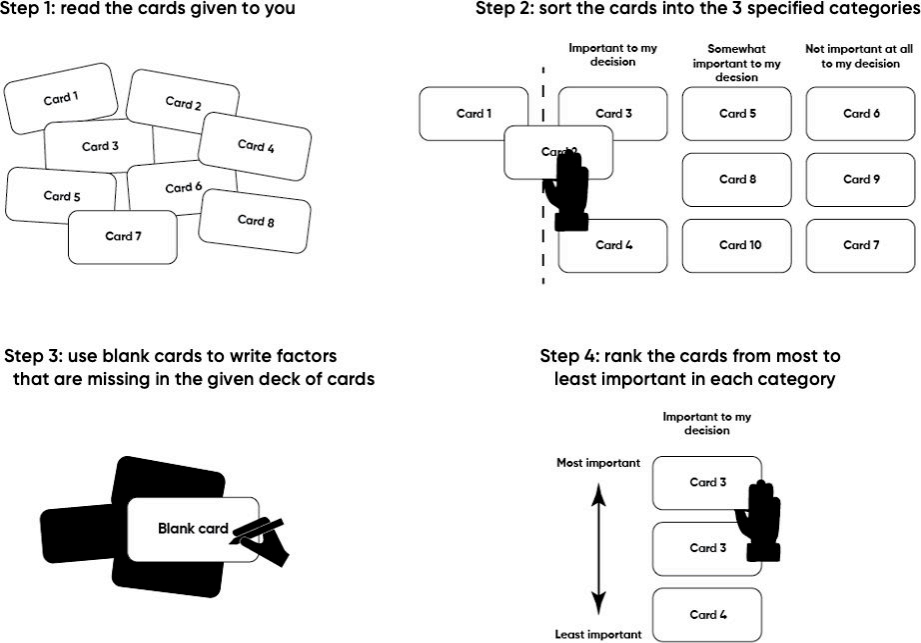
Card sorting task.

Participants will be provided with a briefing sheet outlining the study procedure and a set of standardized instructions before beginning the task. Once participants have reviewed the instructions with study staff, they will be presented with the deck of cards, asked to review them, and sort them into three predefined categories: (1) “important to my decision,” (2) “somewhat important to my decision,” and (3) “not important at all to my decision.” Within each category, participants will be asked to rank cards in descending order of importance. To ensure that a comprehensive list of factors influencing treatment decisions is captured, participants will also be provided with blank cards and markers, allowing them to include additional factors that they perceive as relevant to their decision-making that were not included in the initial deck (Figure 2). Throughout the card sorting activity, participants will be asked to verbalize their thought process to provide “real-time” insights into how they evaluate, prioritize, and consider factors when making a decision regarding rTMS.

Following completion of the card sorting task, a semistructured interview will be conducted to explore the rationale behind their categorizations and rankings, allowing participants to reflect on their decision-making process. The entire session, including the card sorting task and semistructured interview, will be audio recorded for subsequent transcription and analysis, and final card categorizations and rankings will be photographed. Photographs will be taken using research institute iPads (Apple Inc), and no personal identifying information will be captured on audio recordings or photographs (eg, participants’ hands and faces).

#### Patient Surveys

##### Baseline Survey

The baseline survey will assess demographic characteristics (ie, age, sex at birth, gender, educational level, relationship status, employment status, household income, country of birth, race, and religion), clinical characteristics (ie, depressive symptoms, mental well-being, and past treatment use), cognitive flexibility, decisional conflict, and health literacy.

##### Depression

The severity of depressive symptoms will be assessed via self-report using the 10-item version of the Center for Epidemiologic Studies Depression Scale. This brief version of the full 20-item scale has demonstrated good reliability in general (Cronbach α=0.85) and psychiatric (Cronbach α=0.90) samples [30,31]. Individuals rate the frequency of experiencing feelings such as worthlessness and low energy and sleep disturbances over the previous week on a 4-point Likert scale ranging from 0=“rarely or none of the time” to 4=“most of the time.” Total scores range from 0 to 30, with scores greater than 10 indicating significant depressive symptoms.

##### Well-Being

Psychological well-being will be measured using the World Health Organization–Five Well-Being Index [32]. This 5-item scale asks individuals to rate how often, within the previous 2 weeks, they experienced positive mental health states (eg, “I have felt cheerful in good spirits”) on a 6-point Likert scale ranging from 0=“at no time” to 5=“all of the time.” Scores range from 0 to 100 and are calculated by summing the item responses and multiplying the total by 4. Scores below 50 are often used to screen for depression, whereas changes of 10 points across administrations indicate clinically significant pretest-posttest treatment differences (ie, treatment sensitivity) [33].

##### Past Treatment Use

Previous depression treatments will be assessed via a self-report checklist developed for this study, based on American Psychological Association practice guidelines for treating MDD [34]. The checklist includes pharmacotherapy (eg, antidepressants), psychotherapy (eg, cognitive behavioral therapy, interpersonal psychotherapy, psychodynamic psychotherapy, problem-solving therapy, marital and family therapy, and group therapy), herbal supplements, light therapy, acupuncture, ECT, transcranial magnetic stimulation, and vagus nerve stimulation. Participants may also report other treatments not listed.

##### Cognitive Flexibility

Cognitive flexibility, operationally defined as the ability to consider multiple alternatives, adapt to different situations, and think flexibly, will be measured using the Cognitive Flexibility Scale [35]. This scale has demonstrated good internal consistency in general (Cronbach α=0.76-0.77) and clinical anxiety and depression (Cronbach α=0.81) samples [35,36]. Individuals rate their agreement with 12 items on a 6-point Likert scale ranging from 1=“strongly disagree” to 6=“strongly agree.” Total scores are computed by summing responses, with higher scores indicating greater cognitive flexibility.

##### Decisional Conflict

Five dimensions of decision-making will be assessed using the 16-item Decisional Conflict Scale: feeling uncertain, uninformed, unclear about values, unsupported, and ineffective in decision-making. This scale has been widely used as an outcome measure for health decision interventions [37] and has shown good test-retest reliability (Cronbach α=0.78) [38]. Individuals rate their agreement with 16 statements about their treatment decision (eg, opting in or out of rTMS treatment) on a 5-point Likert scale ranging from 0=“strongly disagree” to 4=“strongly agree.” Total scores (0-100) are calculated by summing responses, dividing by 16, and multiplying by 25, with lower scores indicating lower decisional conflict. Subscores for each dimension are calculated similarly by summing the respective subscale items, dividing by the number of items, and multiplying by 25.

##### Health Literacy

The Brief Health Literacy Screening Tool will be used to screen for comprehension and literacy challenges in understanding medical information. It assesses difficulty understanding hospital materials and written and verbal information and confidence in filling out medical forms on a 5-point Likert scale from 1=“always” to 5=“never.” Total scores (4-20) categorize individuals as having inadequate (4-12), marginal (13-16), or adequate (17-20) comprehension. The Brief Health Literacy Screening Tool has shown a sensitivity of 0.79 for detecting inadequate skills and a moderate correlation with other validated literacy tools (*r*=0.40 with the Rapid Estimate of Adult Literacy in Medicine and *r*=0.42 with the Short Test of Functional Health Literacy in Adults), despite consisting of only 4 items [39].

##### Follow-Up Survey

The 6-month follow-up survey will use the same measures as the baseline survey (listed above), with the exception of demographics and past treatment use and the addition of a decisional regret measure.

##### Decisional Regret

The level of distress or remorse following the decision to opt in or out of rTMS treatment will be assessed using the Decision Regret Scale [40]. This validated 5-item measure evaluates regret associated with the effects of a past decision by having individuals reflect on their choice and rate their agreement with statements on a 5-point Likert scale ranging from 1=“strongly agree” to 5=“strongly disagree.” Scores are summed and converted to a scale from 0 to 100 (subtracting 1 from each item and then multiplying by 25), with higher scores indicating greater regret. The tool has demonstrated good internal consistency across clinical samples (Cronbach α=0.81‐0.92) and strong correlations with decision satisfaction (*r*=–0.40 to –0.60) and decisional conflict (*r*=0.31-0.52) [41].

### Chart Review

To understand the clinical implications of patients’ decision-making on rTMS treatments and how past medical treatment and depression history impact decision-making and vice versa, a chart review for each patient will be conducted. Electronic medical records, including consultation notes, progress notes, and patient self-reported outcomes, in Meditech and Greenspace will be accessed to complete this review. Variables of interest include (1) initial decision on rTMS referral, discussions with clinicians, and changes in decision; (2) rTMS treatment and adherence for those who opt in, such as number of appointments attended, number of cancellations or missed appointments, number of rTMS sessions, and length of rTMS sessions; (3) other ongoing treatment for depression, such as current antidepressant use and psychotherapy; and (4) other clinical details, such as hospital admissions and length of patient-provider relationship at HRH.

### Sample Size

As the main aim of this study is to determine what factors are important for patients when making decisions about rTMS as a treatment option in a community setting, sample size calculations were determined based on the minimum number to conduct the card sorting analysis, while considering the feasibility of recruitment (ie, number of referrals based on a treatment capacity of 35 per year). Previous literature has identified that between 10 and 15 participants per group is optimal, with larger card sorting decks requiring larger sample sizes. A total of 24 participants, 12 per group (ie, deciding to opt in vs opt out of rTMS treatment), are required for analyzing the data from a 24-card deck. Previous experience from our research team indicates that a 20% attrition rate is expected at the 6-month follow-up in a sample of individuals with depression [42]. Therefore, a total of 30 participants will be recruited.

### Data Analysis

#### Factors Influencing Patients’ Decisions on rTMS Treatment at Baseline

Hierarchical agglomerative clustering will be conducted independently for each study cohort (ie, those who opt in vs out of rTMS treatments) to identify patterns in how participants prioritize the factors that influence their decision-making at baseline. The analysis will use 2 data points collected for each card in the 24-card deck plus any blank cards on which participants write personal factors: the category (ie, the pile that the card is placed in: important=1, neutral=2, and not important=3) and the rank of the card within the category (with 1 being the most important, followed by 2, 3, and so on). A maximum of 52 variables will be included in the cluster analysis assuming that each participant adds only 2 personalized factor cards. The analysis will use the squared Euclidean distance to calculate the distance between observations, and the Ward method will be applied to cluster card rankings and groupings, minimizing variance within clusters. The resulting dendrograms will be examined separately for those who choose to opt in vs opt out of rTMS treatments. The cophenetic correlation coefficient will be used to assess how well each dendrogram preserves the original squared Euclidean distances, as suggested in the literature [43]. To determine the optimal number of clusters for each group, the dendrogram will be cut at different levels, and silhouette scores will be calculated for k-cluster solutions. The number of clusters with the highest average silhouette score will be selected, and the final dendrogram will be presented for visualization.

Qualitative data from the card sorting baseline interviews will be analyzed to provide context and deeper insights into the clustering results. A CCA will be used to identify relationships between key factors identified as important, neutral, or not important for making decisions about rTMS treatment. Qualitative data will be mapped onto the cluster groupings to both interpret clusters and conceptually validate groupings. Specifically, participants’ explanations, reasoning, and thought process during card grouping and prioritizations will be used to define clusters, refine cluster interpretations, and support the selection of the final number of clusters. While the analysis will be conducted using CCA methodology [44], it will be guided by 2 key frameworks—bounded rationality and prospect theory—rather than grounded theory [45]. Bounded rationality will inform the understanding of how participants make decisions within cognitive and informational limits [46], whereas prospect theory will provide insights into how patients weight potential gains and losses [47] from the reference point of being diagnosed with TRD. The analysis will follow a three-step process of analyzing transcripts, with memoing throughout the process providing further context: (1) open coding of each interview and internal comparisons to develop categories and understand the core decision-making process; (2) axial coding to examine relationships among codes and identify properties and dimensions within categories, with a focus on differences and similarities between those who opt in vs opt out of rTMS treatment; and (3) triangulation of data across interview groups to validate and refine themes and concepts that have been identified.

#### Decisional Changes, Conflict, and Regret

To examine changes in participants’ priorities for decision-making on rTMS treatment, 2 analyses will be conducted. First, the hierarchical clustering analysis performed at baseline will be repeated using data from participants’ 6-month card sorting task. This will allow for the visual comparison of dendrograms created for the baseline and 6-month card sorting activities, allowing for the assessment of how participants’ prioritization of decision-making factors evolves over time. Differences in clustering patterns between baseline and the 6-month follow-up will be examined to determine whether shifts occur in the grouping of decision-making factors. Additionally, subgroup analyses will be conducted for those who opt in to rTMS to account for participants’ treatment status (ie, whether they have started, completed, or are on a waitlist for rTMS treatment), ensuring that observed changes are contextualized within each individual’s phase of treatment. Second, changes in participants’ card rankings (ie, sorted by importance category and rank within each category) from baseline to the 6-month follow-up will be analyzed qualitatively using a longitudinal adaptation of the CCA. This approach will build on the baseline CCA by comparing thematic patterns across time points. Specifically, the three-step CCA process will be extended as follows: (1) open coding of 6-month interviews to identify emergent themes and changes in decision-making perspectives; (2) axial coding to examine relationships among new and preexisting codes, focusing on whether shifts occur within or between importance categories and rank orderings; and (3) triangulation across baseline and 6-month interview data to validate and refine evolving themes, ensuring consistency and reliability in interpretations of decisional change.

In addition to evaluating how factors influencing rTMS decisions change over time, group differences in individual-level variables between those who opt in vs out of rTMS will be examined. Exploratory analyses could include the use of chi-square tests and *t* tests to examine differences in demographic characteristics, clinical characteristics, and psychological constructs (ie, cognitive flexibility and decisional conflict) between those who opt in vs out of rTMS treatment. In addition, correlations between baseline cognitive flexibility and decisional conflict and decisional regret at 6 months will be explored. Finally, for those who opt in for rTMS treatment, changes in cognitive flexibility and decisional conflict from baseline to 6 months will be examined to understand how treatment impacts psychological constructs or vice versa. Due to the small sample size, exploratory quantitative analyses will primarily serve to guide future research hypotheses.

All quantitative statistical analyses will be conducted using SPSS (version 29.0; IBM Corp), and qualitative data analyses will be performed in NVivo (version 14; Lumivero).

### Data Management and Governance

All collected data (ie, survey responses, interview transcripts, audio recordings and files, photographs of sorted cards, and qualitative memos) will be stored in the hospital’s secure OneDrive (ie, no data will be stored on local computers or outside of the institutionally secure OneDrive).

Separate (1) master linking logs containing patients’ study IDs and personal identifying information and (2) contact datasets containing participant-provided and consented contact information for future research will be kept as password-protected files and stored on HRH’s institutionally secure OneDrive. These files will be stored in different folders and will only be accessible to authorized research team members. Upon completion of the study, all master linking logs will be deleted. Contact datasets will only be retained for 7 years, after which they will be destroyed by the study team in accordance with the HRH data retention policy. Contact information will only be used for recontacting participants for future studies that have received research ethics board approval.

## Results

This study was funded by a charitable donation from Jack and Pat Kay to Humber River Health, which is also supporting the establishment of the rTMS clinic, committed to in April 2024. Recruitment commenced in December 2025 and is expected to conclude in December 2026; no participants have been enrolled as of February 2026.

## Discussion

This will be the first study, to our knowledge, that aims to explore factors influencing patient decision-making regarding rTMS treatment in a community hospital clinic. While previous research has focused on posttreatment perceptions, this study will prospectively examine patients’ decision-making processes at the time of referral, providing a real-time perspective on how individuals evaluate rTMS before undergoing treatment. Additionally, by exploring patients’ reflections on their decisions 6 months later, this study will also illustrate what factors contribute to decisional regret over time for patients with TRD. By considering both those who opt in for and those who decline rTMS treatment, this study will provide a comprehensive understanding of the motivations and barriers that shape treatment choices among individuals with TRD for unfamiliar treatment options such as rTMS.

Given the growing interest among health care providers in understanding the effectiveness and accessibility of rTMS at the community level, it is important to explore factors that drive patients’ decision-making. Such insights could inform the development of patient-centered education and engagement strategies, which can help enhance both patient and public knowledge of rTMS. Furthermore, this study will contribute to existing models of health decision-making for depression by investigating the role of individual differences in cognitive flexibility, health literacy, and decision-making ability on treatment choice. The findings will inform patient-provider communication strategies, enhance shared decision-making models, and better equip clinicians to support patients in making informed decisions about rTMS.

A key strength of this study is the mixed methods design, which combines quantitative (ie, surveys and chart reviews) and qualitative (ie, card sorting interviews) data to capture a comprehensive view of what personal factors influence rTMS decision-making. This approach will allow us to have a more complete understanding of the facilitators of and barriers to rTMS treatment, potential associations between different factors, and how individual-level differences all play a role in the treatment decision-making process for rTMS. By examining decision-making at the time of referral and following patients over time, this study will offer novel insights into how patients evaluate rTMS initially and how their perceptions of it change over time. In addition, the focus on both those who opt in and out of rTMS provides a more balanced view of the decision-making process for patients overall.

Despite the strengths and novelty of this study, there are a few limitations to consider. First, as rTMS is not publicly funded across Canada and public awareness of the treatment remains low, patient decisions may be strongly influenced by the amount and type of information provided about rTMS by the referring physician or clinic rather than by independent preexisting attitudes. Second, this study is being conducted in a newly established clinic, which may introduce variability into patient experience. For example, factors such as evolving clinic procedures, staff expertise, and ongoing improvements to the clinic workflow and procedures could influence perceptions and decision-making. Finally, while the study focuses on patient-level factors, broader health care system–level influences such as referral pathways, institutional policies, and other system-level factors that can influence patient decision-making indirectly will not be examined.

## Abbreviations

CCA: constant comparative analysis
CONSORT: Consolidated Standards of Reporting Trials
ECT: electroconvulsive therapy
HRH: Humber River Health
MDD: major depressive disorder
rTMS: repetitive transcranial magnetic stimulation
TRD: treatment-resistant depression
